# Analyzing the Drivers of Household Dietary Diversity: Evidence from Burkina Faso

**DOI:** 10.1177/03795721211029092

**Published:** 2021-09-01

**Authors:** Arkadeep Bandyopadhyay, Beliyou Haile, Carlo Azzarri, Jérôme Somé

**Affiliations:** 1 8357 International Food Policy Research Institute, Washington, DC, USA; 2 1810 Tufts University, Medford, MA, USA

**Keywords:** dietary diversity, production diversity, measurement, Burkina Faso

## Abstract

**Background:**

The diets of millions of poor individuals lack adequate amount of essential nutrients.

**Objective:**

To examine the determinants of household dietary diversity in Burkina Faso and assess whether the choice of a diversity metric matters.

**Methods:**

Using survey data from 2014, we construct 3 metrics—Household Dietary Diversity Score (HDDS), Berry Index (BI), and Healthy Food Diversity Index (HFDI). Unlike the oft-used HDDS, the BI captures the quantity distribution of food items while the HFDI captures all 3 aspects of a healthy diet—count, quantity distribution, and health value. We fit linear (for BI and HFDI) and Poisson (for HDDS) models controlling for several socioeconomic and climatic covariates.

**Results:**

Some parameter estimates are sensitive to the diversity metric with fewer significant covariates observed in the HFDI model. Overall, diets are more diverse for households in urban areas, with female or better educated heads, with higher asset-based wealth and with more diverse on-farm production, while remoteness reduces dietary diversity. Higher precipitation seems to reduce diversity, potentially driven by the spatial heterogeneity in precipitation and on-farm production diversity.

**Conclusions:**

The sensitivity of estimates to the metric used underscores potentially more complex interactions that determine the quantity distribution of food items consumed. Policies that enhance on-farm production diversity, market access, and women’s empowerment may help improve dietary diversity and subsequent nutritional benefits. Efforts should be made to compile health value data that are relevant to developing countries facing nutrition transition.

## Introduction

In many poor countries, both production and consumption are heavily reliant on cereals for which production is insufficient to meet domestic caloric needs. While the nutritional composition of these staples may vary depending on the variety, they generally lack crucial micronutrients such as vitamins A and C, and bioavailability of some of the B vitamins and minerals (eg, vitamin B6, iron, zinc) may be limited.

The importance of nutritional adequacy for the growth, development, and maintenance of bodily functions has been comprehensively established. Although subjective perceptions of a healthy diet vary across regions^1^ and over time,^2^ a shared vision is that it comprises a wide variety of foods in correct amounts and proportions.^3^ More diverse diets are generally positively correlated with the mean adequacy ratio (note 1), indicating nutrient adequacy as well as nutritional outcomes such as height-for-age z-score among children.^4,5^ On-farm production diversity is among the food-based strategies pursued to enhance dietary diversity and intake of essential nutrients.

Although several dietary indicators measuring diversity exist, data availability limits which ones can ultimately be utilized. There is a positive relationship between precision and cost in collecting food consumption-based surveys^6^ and the latter becomes a prohibitive factor when collecting data in a developing country. Thus, in the absence of high-quality individual-level consumption data, researchers often need to rely on nutritional information at the household level from relatively more abundant Household Consumption and Expenditure Surveys.^7^

This study examines the association between household dietary diversity and various household-level socioeconomic and landscape-level climatic factors in Burkina Faso using alternative indicators of household dietary diversity. Household-level factors such as household head characteristics, wealth, location of residence (rural vs urban as well as region), and agricultural production can affect the dietary diversity through their impact on food availability from own production, food purchasing power, access to food markets, and intrahousehold decision-making. Given the dominance of rainfed agriculture in the study setting, climatic factors such as precipitation and temperature that shape agricultural production patterns will affect the quantity and quality of food available for consumption.

Food consumption data quality, unit of reporting, dietary metrics used, as well as the reference period are all crucial elements of a nutrition analysis.^8-10^ When data are collected at the household level, for example, it is difficult to draw inference about individual-level outcomes without making assumptions about intrahousehold redistribution. Any empirical analysis assuming equitable distribution determined by caloric needs based on either per-capita or per-adult equivalent (AE) values could produce biased results, since intrahousehold inequality has been documented across several countries,^11^ especially for vulnerable groups that are more likely to receive a smaller share of resources.^12^ Although individual-level disaggregation through per capita or AE calculations offers an easy solution to this problem, the level of accuracy varies on a case-by-case basis.^13-15^ In this study, we rely on household-level food consumption data only, leaving aside the potentially important concern of intrahousehold food redistribution.

One of the most commonly used indicators of dietary diversity is the Household Dietary Diversity Score (HDDS) that sums the number of distinct food groups consumed by the household within a specific reference period. Although the HDDS is easy to calculate and interpret, as the same weight is assigned to different food groups regardless of their health contribution or quantity consumed, it has not been validated as a measure of nutritional adequacy. There is a substantial nutritional difference between consuming a small portion of vegetables and a large portion of cereals that is not captured by the HDDS.

Considering these shortcomings, we analyze and contrast the correlates of household-level dietary diversity in Burkina Faso using 3 distinct measures of diversity: the HDDS, the Berry Index (BI), and the Healthy Food Diversity Index (HFDI). The BI builds upon the HDDS by accounting for the quantity of each food group consumed, with the HFDI taking the BI a step further by associating a health value to each food group consumed. Although we would expect both the BI and the HFDI to be marked improvements over the HDDS, our regression estimates indicate that they are indeed quite similar, although with some interesting distinctions.

Our article contributes to the literature on dietary diversity by contrasting the oft-used HDDS with 2 less frequently used indicators of diversity; the BI and HFDI. We demonstrate how the BI and HFDI can provide more accurate measures of dietary diversity. Additionally, our estimates show a significant positive association between market access and household dietary diversity, highlighting the importance of investments that enhance smallholders’ access to local markets and foods not grown on their own farms.

The rest of the article is organized as follows. Previous Literature section covers the existing studies on the topic; Data and Methods section presents the data used, including construction and interpretation of the diversity indicators used; Statistical Model section presents the statistical model we estimate; Results and Discussion section discusses the study finding; and, finally, Conclusion section concludes the article.

## Previous Literature

According to the World Health Organization, a healthy diet consists of fruits, vegetables, legumes, nuts, and whole grains.^3^ Since different food items and groups are good sources of various macro- and micronutrients, a diverse diet is generally positively correlated with nutrient adequacy.^16^ Although diversity is a critical aspect of dietary quality, it does not guarantee a balanced diet since disproportionately low or high amounts of energy from a given macronutrient may be a sign of underconsumption (disproportionately high amount of total carbohydrates) or overconsumption (disproportionately high amount of lipids, and sometimes proteins).^17^

Existing evidence shows that individual-level dietary diversity score (DDS) is positively correlated with micronutrient adequacy,^18^ with child height-for-age z-scores (linear growth),^4,5^ as well as with overall health of the poor, especially poor women.^19^ Given that individual-level food consumption data collection is more resource intensive than household-level collection, many large-scale surveys gather household-level data for subsequent analysis of household-level dietary indicators including the HDDS. Although the HDDS is a good proxy for household socioeconomic status, a systematic review of studies based on DDS and HDDS shows that HDDS can be more affected by measurement bias than the DDS.^20^

One improvement over the HDDS is the BI^21^ that accounts for potentially polarized consumption on some selected food groups. The BI, originally used to measure corporate diversification, is defined as $1-\overset{n}{\underset{i=1}{\sum }}p^{2}$ , where, in our case, *p_i_* is the ratio of household’s consumption of food item *i* to its total consumption of food group *n*. However, as a general index of diversity, it was later used in studies relating to food diversity.^22,23^ In a US study using the BI, researchers show that both variety and amount of vegetables are important to prevent coronary heart disease.^24^ In another study in Nigeria, a positive association between the BI and the likelihood of a household meeting caloric, protein, and micronutrient requirements is found.^25^ In a multiethnic analysis of the determinants of atherosclerosis, the BI was found to be weakly positively correlated with diet quality proxied by the Alternative Healthy Eating Score.^26^

Although the BI addresses the issue of distribution or relative intensity in the consumption of food groups, it still allocates equal *nutritional* weight to the different food groups. Observing this limitation, Drescher et al^27^ propose the HFDI that assigns a *health factor* to each food group to better capture the nutritional and health implications of diverse diets. The authors used dietary guidelines for Germany (German Nutrition Society—DGE), where a healthy diet should comprise 73% of plant-based food, 25% of animal-based foods, and 2% of fats and oils. These percentages refer to the quantity of foods consumed and have been used by researchers to examine the health implications of diverse diets in other settings,^28^ although DGE’s recommendations may not adequately capture the local context in Burkina Faso.

To empirically examine the determinants of household dietary diversity using the HFDI and nationally representative data, country endorsed dietary recommendations are needed, despite being often missing as in the case of Burkina Faso. The missing information would usually require researchers to use proxy data from other comparable countries or extrapolate using alternative data sources, such as subnational data. The evidence from developed countries shows that the HFDI is negatively associated with the risk of metabolic syndrome among certain ethnic groups^29^ and positively associated with nutrient adequacy.^30^ Other clinical research in the United States shows a negative association between the HFDI and body adiposity suggesting that a more diverse healthy diets protect against excess adiposity.^31^

Several socioeconomic factors are found to be significantly correlated to dietary diversity, including household sociodemographic and economic status, on-farm production diversity, intrahousehold gender relationships, and market access. Forshee and Stodrey^32^ find that family income is positively associated with a healthy diet, while better household socioeconomic status is also found to increase the share of energy derived from fat.^33^ The role of agricultural income in improving dietary quality has also been shown in other settings.^34^

In Burkina Faso, better educated household heads have enjoyed more diverse diets both at the household and member level.^35^ Evidence from other poor countries also shows a positive association between female education and dietary quality and a negative association between female household headship and dietary quality after controlling for total household consumption expenditure.^36^ Female farmers and female-headed households often have limited access to productive resources (eg, land, capital, and credit) and are concentrated among the poorer segments of the society with implications for their agricultural productivity and on-farm diversity.^37^

Finally, in settings where subsistence production accounts for a significant share of households’ food and caloric consumption, on-farm diversity can contribute to dietary diversity. Indeed, a positive association has also been documented between household dietary quality and production diversity in Malawi,^38^ as well as Indonesia, Kenya, and Ethiopia.^39^ At the same time, smallholders rely on local markets to acquire some of the food they consume, which explains the positive association between market access and dietary diversity documented in several studies.^40-44^

## Data and Methods

### Setting

Burkinabe population is overwhelmingly poor and rural, conducting an economy dominated by rainfed agriculture. In 2012, average per capita income was $460, and 44% of the population lived under $1.90 (in purchasing power parity) per capita/day.^45^ Although poverty rate has markedly decreased (from 81.6% in 1998 to 43.7% in 2014^46^), considerable efforts are still needed for the country to achieve Sustainable Development Goal 1 (SDG 1) on poverty eradication by 2030.^47^ Undernutrition is also rampant in Burkina Faso, making it hard for the country to achieve SDG 2—eradication of hunger and all forms of malnutrition by 2030. The country has experienced only 4 percentage points reduction in the prevalence of stunting among children between 1993 and 2010 (from 38.8% to 34.6%), while the prevalence of child wasting and underweight has remained mostly constant (at around 16% and 25%, respectively) during the same period, according to the DHS data.^48^ More than a fifth of Burkinabe population (21%) was undernourished between 2015 and 2017.^49^ These trends underline the need to identify potential factors that can contribute to better dietary, nutritional, and health outcomes.

### Data Sources and Variables

We use data from the 2014 Burkina Faso Continuous Multisectoral Survey (*Enquête Multisectorielle Continue*; EMC 2014) conducted between January and December 2014. Data are nationally representative and were collected from all 45 provinces. A 2-stage sampling technique was applied, with the first stage involving a random sampling of 905 enumeration areas (EAs) using probability proportional to the number of households in the EA, and the second stage involving random sampling of 12 households per EA. A total of 10860 households (note 2) were included in the EMC with food consumption data collected across 4 seasons that correspond with the different stages of the agricultural production cycle (note 3).

During each visit, the most knowledgeable household member in charge of food acquisition and processing was asked to report household-level consumption of various food items in the previous 7 days. Although consumption quantity and expenditure data were collected for all 4 periods, food consumption data from only the first visit were publicly released and, as a result, we are unable to examine seasonality in household food consumption. However, several studies^10,13,50^ show the usefulness of food consumption data from whole household consumption expenditure surveys like the EMC for food security and nutrition analysis, despite being based on a 7-day recall period and collected at 1 point in time during the agricultural season.

In addition to microdata from EMC, we use province-level data on precipitation and temperature.^51^ We compute mean and coefficient of variation (CV) of monthly values for 2000 to 2013, as well as 2013 cropping season (June through December) matching the reference cropping cycle in the 2014 EMC.

### Index Construction

Using the household-level food consumption data and trying to capture dimensions of dietary diversity beyond the simple count of food groups based on the HDDS, we construct 2 additional household-level indices of dietary diversity.

#### Household Dietary Diversity Score

Following Swindale and Bilinsky,^52^ we define the HDDS based on the consumption of the following 12 food groups: cereals; roots, tubers and plantains; pulses, legumes, nuts, and seeds; vegetables; fruits; meat; fish and seafood; milk and dairy products; eggs; oils and fats; sugar/honey; and miscellaneous. A more diversified household diet is found to be positively correlated with caloric and protein adequacy, share of protein was obtained from animal-sourced foods and household income.^52,53^

#### Berry Index

Although the HDDS has widely been used in the literature, it does not consider the relative quantities of the different food groups consumed. To illustrate the potential problem with this approach, we can consider the following hypothetical consumption set for household # 1  (listed in food item—*food group* format): rice—*cereals* (600 g), potatoes—*roots* (250 g), beans—*legumes* (100 g), spinach—*vegetables* (100 g), apples—*fruits* (100 g), and tilapia—*fish and seafood* (5 g). The HDDS for this household would be 6. However, we notice that this household consumes a relatively small amount of nutrient-rich tilapia and large amount of rice that is not captured by the indicator. Given the polarization in consumption, a more accurate measure of dietary diversity would consider the relative amounts consumed.

Building on the shortcomings of HDDS, the BI^21^ is a useful indicator that controls for the actual quantities of individual food items consumed by the household. For a household *k*, the BI is defined as follows:


1 $$BI_{k}=1-\;\sum s_{i}^{2}$$

where *s_i_* = (quantity of a food item *i*)/(quantity of all food items)   (food items consumed by each household k) . According to this formulation, the measurement units of numerator and denominator should be expressed in the same metrics (eg, grams). Following our example above, the BI for the same household would be:


$$BI_{1}=1-({\left(\frac{600}{1155}\right)}^{2}+{\left(\frac{250}{1155}\right)}^{2}+{\left(\frac{100}{1155}\right)}^{2}\;+{\left(\frac{100}{1155}\right)}^{2}+{\left(\frac{100}{1155}\right)}^{2}+{\left(\frac{5}{1155}\right)}^{2})$$

$$BI_{1}≅0.66$$

Now, consider the hypothetical consumption set for another household #2: rice—*cereals* (550 g), potatoes—*roots* (230 g), beans—*legumes* (100 g), spinach—*vegetables* (100 g), apples—*fruits* (100 g), and tilapia—*fish and seafood* (75 g), for which the BI is shown below:


$$BI_{2}=1-\;\left({\left(\frac{550}{1155}\right)}^{2}+{\left(\frac{230}{1155}\right)}^{2}+{\left(\frac{100}{1155}\right)}^{2}\;+{\left(\frac{100}{1155}\right)}^{2}+{\left(\frac{100}{1155}\right)}^{2}+{\left(\frac{75}{1155}\right)}^{2}\right)$$

$$BI_{2}≅0.71$$

For the same level of total consumption, simply increasing the consumption of *tilapia* by 70 g (by reducing the consumption of rice and potato by, respectively, 50 g and 20 g) yields a higher value of the BI, while the HDDS is left unchanged. Being able to weigh the relative proportions of each food item allows us to draw a more complete picture of the diversity in the diet using the BI. However, while the BI for household #2 is higher than for household #1, we cannot conclude that a higher BI value necessarily implies a healthier diet without accounting for the nutritional and health value of different food items.

#### Healthy Food Diversity Index

The third index used—HFDI—assigns different weight to different food items based on their health value. The HFDI for household *k* is computed as follows:


2 $${\text{HFDI}}_{k}=\;\left(1-\;\sum s_{i}^{2}\right)hv_{k}$$

where hv is the health value of the food item defined as: $\sum hf_{j}s_{i}$ ; where *s_i_* is as defined before, $hf_{j}$ is the health factor for food group *j* as calculated in the next section. The share of food item *i* is multiplied by the heath factor of the food group *j* to which it belongs; for example, $s_{\text{rice}}$ would be multiplied by $hf_{\text{cereals}}$ . The values of both the BI and HFDI range between 0 and 1. As with the BI, it is difficult to determine an optimum value of the HFDI, as it depends on the specific consumption set based on which the health factors are determined, in addition to the relative quantities of food items consumed. For example, it is possible that a household consumes only the food items associated with the highest health factors driving up the health value of the diet but, because of the polarized diet favoring only certain items over others, the HFDI would fall—as would the BI—due to the relatively low diversity in consumption. The BI is positively correlated with HDDS and the HFDI is positively correlated with *both* HDDS and dietary health value. However, different values of the HFDI can be compared only if they have been calculated based on the same health factors and consumption set.

### Health Factors

The HFDI is based on the idea that every food group is associated with a constant health factor, for a given consumption set. These health factors are determined based on a recommended consumption set associated with positive health outcomes along with adequate nutrient intake. Given that national food-based dietary guidelines are unavailable for Burkina Faso, we rely on a self-reported consumption set based on the 75th consumption percentile that has been found to meet micronutrient adequacy among women of reproductive age from 2 districts of the nation’s capital, Ouagadougou (note 4),^54^ our reference consumption set. There are obvious caveats from the use of these data, covering dietary data for a specific female age-group who reside in an urban area. Although, as we will show, the absolute value of consumption of each food group is not a concern as we are using the relative shares of each food group.

Table 1 shows that, based on the reference consumption set, the imputed “recommended” diet is comprised of 85% of plant foods, 13% of animal foods, and 2% of oils and fats. Although this diet appears to be different from the German recommendations, our values of interest are solely the health factors. Additionally, the food groups identified as beverages and miscellaneous (eg, beer, coffee, tea, spices that are not shown in Table 1) have been omitted from this exercise and as such are not associated with any health factors, given their negligible positive impacts on the human body. We do not expect their omission to significantly bias our results.

**Table 1.** Recategorization Based on HDDS Food Groups and Health Factor Calculations.

Food Groups	75th Percentile (g/d)^a^	Food category	Food category		Food group proportion^d^	Health factors^e^
			G^b^	Proportion^c^		
Grains (cereals)	662	Plants foods	1636	0.85	0.40	0.34
Roots, tuber, plantains	150				0.09	0.08
Pulses, legumes, nuts, and seeds	170				0.10	0.09
Vegetables	294				0.18	0.15
Fruits	360				0.22	0.19
Meats	84	Animal foods	247	0.13	0.34	0.04
Fish and seafood	65				0.26	0.03
Milk and dairy products	38				0.15	0.02
Eggs	60				0.24	0.03
Oils and fats	40	Oils and fats	40	0.02	1.00	0.02
Total (grams)	1923		1923			

Abbreviation: HDDS, Household Dietary Diversity Score.

^a^ Information on quantity of consumption has been sourced from Arimond et al.^54^

^b^ Calculated as the total quantity of the relevant food category. For example, for the case of plant foods, it is the sum of grains, roots, tuber, plantains, pulses, legumes, nuts and seeds, vegetables, and fruits.

^c^ Calculated as the ratio of the total quantity of each food category to the total quantity across all food categories. For example, for plant foods, this ratio is: (1636/1 923) ≈ 0.85, where 1636 is the total quantity of plant foods and 1923 is the total quantity of food across all food categories.

^d^ Calculated as the ratio of the quantity of individual food groups within a food category to the total quantity of the food category. For example, for cereals, this would be calculated as: (662/1636)≅0.4, where 662 is the total quantity of cereals and 1636 is the total quantity of plant foods.

^e^ Health factors are calculated as the product of the food category proportion × the food group proportion. For example, for grains it would be: 0.85×0.40=0.34 .

## Statistical Model

The following model is estimated to assess the determinants of household dietary diversity:


3 $$y_{i}=f\left(X_{i},Z_{i}\right)+e_{i}$$

where *i* is the index for household; *y* is each of the 3 dietary indicators defined above; matrix *X* consists of household-level variables defined below; *Z* includes province-level weather variables; and *e* is the model error. Guided by the research discussed in Previous Literature section, household-level covariates we control for include household size, area of residence (urban versus rural), gender and education level of the household head, number of durable agricultural and nonagricultural assets owned by the household, household crop production diversity measured using the number of unique plant-based food groups grown by the household (note 5), and indicators of travel time from the household’s residence to the nearest market (note 6). Matrix *Z* includes precipitation and temperature variables defined in the Data Sources and Variables section. We first estimate the model controlling only for household-level covariates and then controlling for both household-level and climatic variables as a robustness check.

Since the BI and HFDI are continuous indicators ranging between 0 and 1 and HDDS is a count variable with values ranging between 1 and 12, we fit Equation 1 using ordinary least squares when *y* is the BI or the HFDI and a Poisson model when *y* is the HDDS. Estimates control for multistage clustered sampling design, with robust standard errors clustered by EA.

## Results and Discussion

### Descriptive Summary

Looking at the full sample, we see that study households have approximately 7 members and more than 70% of households are located in rural areas (see Table 2). Regarding agricultural production, we find that households produced 1.2 different food groups and 2.3 food items, on average. Only 0.2% and 0.8% of households produced fruits and roots, tubers and plantains, respectively. In contrast, cereals are produced by 68% of households; while pulses, legumes, nuts, and seeds are produced by 49% of households. Households earned about 20,000 CFA (approximately 34 USD) annually from nonfarm activities with more than 30% of household food consumption coming from own production (note 7). More than 80% of households can access a market within 60 minutes of travel from their residence.

**Table 2.** Descriptive Summary.^a^

Variable	Mean or %	SD	Min	Max
*Household level*				
Household size	6.9	4.0	1.0	23.0
Household head age (years)	46.1	15.4	15.0	99.0
Female-headed households (%)	13.9%			
Household head education				
None (%)	75.2%			
At least primary (%)	24.7%			
Urban households (%)	27.9%			
Total land area owned (hectare)	2.4	2.9	0	18.5
*Agricultural production*				
Number of food groups produced^b^	1.2	1.0	0.0	4.0
Number of food items produced	2.3	2.0	0.0	10.0
Household produces cereals	67.5%			
Household produces roots, tubers, and plantains	0.8%			
Household produces pulses, legumes, nuts, and seeds	48.9%			
Household produces vegetables	6.0%			
Household produces fruits	0.2%			
Total production quantity (kg)	278.9	361.5	0	2500
*Household assets and income*				
Number of nonagricultural durable assets^c^	4.7	3.7	0	22.0
Number of agricultural durable assets^d^	2.0	1.9	0	10.0
Household off-farm income (‘000 CFA)	20.2	71.6	0	100.9
Share of food consumed from own production (%)^e^	31.5	28.4	0	100.0
*Travel time to the nearest market (%)*				
0-14 minutes	37%			
Above 15 minutes and less than 60 minutes	46%			

Abbreviations: CV, coefficient of variation; EMC, *Enquête Multisectorielle Continue*; Max, maximum; Min, minimum; SD, standard deviation.

^a^ Results have been weighted by survey sampling weights. For education level, having no education has been combined with preschool education level. CV means coefficient of variation. CFA is Burkina Faso’s currency with an exchange rate of about 520 CFA per one US dollar in the year in which EMC was conducted (2014).

^b^ The grouping of food items produced by households is in line with the 12 food groups defined in Swindale and Bilinsky^52^—cereals, roots, tubers, and plantains; pulses, legumes, nuts and seeds; vegetables; fruits; meat; fish and seafood; milk and dairy products; eggs; oils and fats; beverages; and miscellaneous. Since the EMC did not collect data for livestock holdings, our grouping is based only on plant-based items produced by the households and the “miscellaneous” category.

^c^ Durable nonagricultural assets include automobile, motorcycle, cycle, radio, solar plate, VCR/DVD, television, hi-fi system, computer, air conditioning, refrigerator, antenna with decoder, mobile telephone, landline telephone, freezer, gas/electric cooker, improved fireplace, electric iron, charcoal iron, fan, generator, complete dining table, bed, mattress, complete living room, and buffet.

^d^ Durable agricultural assets include rice huller, plough, cart, milling machine, seeder, tractor, sprayer, rototiller, multicutter, hoe, plough animals, corn sheller, rice thresher, millet thresher, pump unit, hand pump, rocker, baler, straw axe, reaper, fertilizer spreader, and other equipment.

^e^ Based on consumption quantity in the last 7 days

Summary of food consumption data based on a 7-day recall shows that almost all households consumed cereals and vegetables, while 80% reported consuming fish and seafood. In contrast, only 16% consumed roots, tuber, and plantains; 12% consumed fruit; and only 6% consumed eggs (see Table A1). The relatively low consumption of these nutritious food groups suggests that the target population is likely not getting adequate nutrients that are essential for the health and proper functioning of the human body.^55,56^

**Figure A1.**
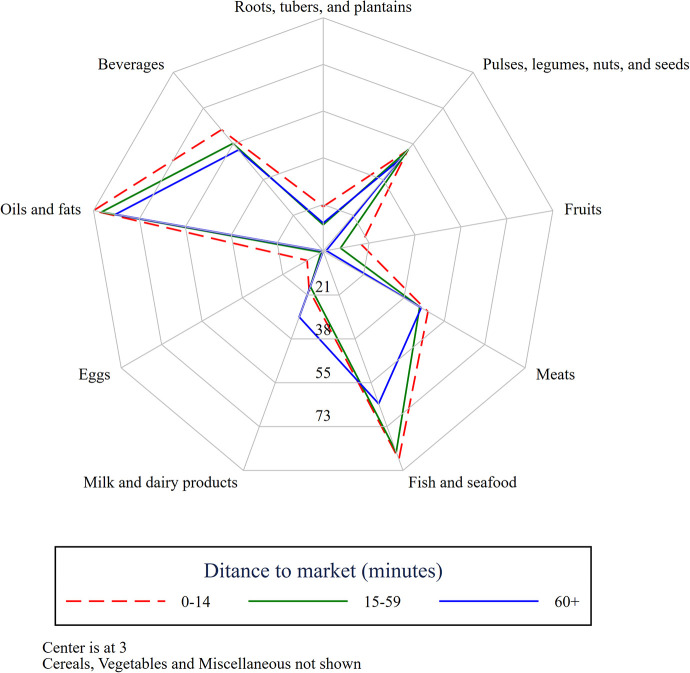
Food group consumption.

Although the relationship between food-group consumption and distance to the nearest market is not consistent across all food groups, we find that proximity to markets is positively associated with the consumption of eggs, fruits, meats, and roots, tubers, and plantains (see Figure A1). Summary of crop production shows that fruits and roots, tubers, and plantains are grown by the smallest share of households thereby limiting their consumption, especially among households with limited access to markets. As noted, consumption rates for these food groups are higher among households that are closer to a market, potential explanation for the observed positive association between market proximity and consumption only for some food groups.

The average value of HDDS is approximately 6.8 (Table 3), while the BI and HFDI are on average 0.6 and 0.2, respectively. The minimum of zero of the BI is due to households reporting the consumption of just one food item (note 8), food items for which unit information is missing (*feuilles* — *oseilles, baobab, boulvaka*, and *beurre de karité*), or food items for which edible quantity factor is missing in the West Africa Food Consumption Table (*kapok — voaga*). Consequently, the HFDI (which is the product between the household BI and dietary health value) is also zero for the same households. Overall, there are 78 households in our sample with null value in the BI and HFDI.

**Table 3.** Descriptive Summary of Dietary Diversity Indicators.^a^

	*Panel A: Summary Statistics*			
Indicator	Mean	SD	Min	Max
HDDS	6.8	1.8	.01	12.0
Berry Index	0.6	0.2	0	0.9
HFDI	0.2	0.1	0	0.3
	*Panel B: Correlation coefficients*			
	HDDS	Berry Index	HFDI	
HDDS	1			
Berry Index	0.58	1		
HFDI	0.41	0.87	1	

Abbreviations: HDDS, Household Dietary Diversity Score; HFDI, Healthy Food Diversity Index; Max, maximum; Min, minimum; SD, standard deviation.

^a^ Pair-wise correlations are all significant at the 1% level.

Although the BI and the HFDI embed additional dimensions over the HDDS, Table 3 confirms that these indicators are positively correlated with each other. The HFDI and BI are highly correlated because the HFDI is built on the BI by construction. However, the HDDS and HFDI have a relatively lower level of correlation. This result suggests that the simple increase in the number of food groups consumed does not necessarily translate into a better diet; the added food group must be consumed in an adequate quantity and should provide a substantial nutritional value.

**Figure 1.**
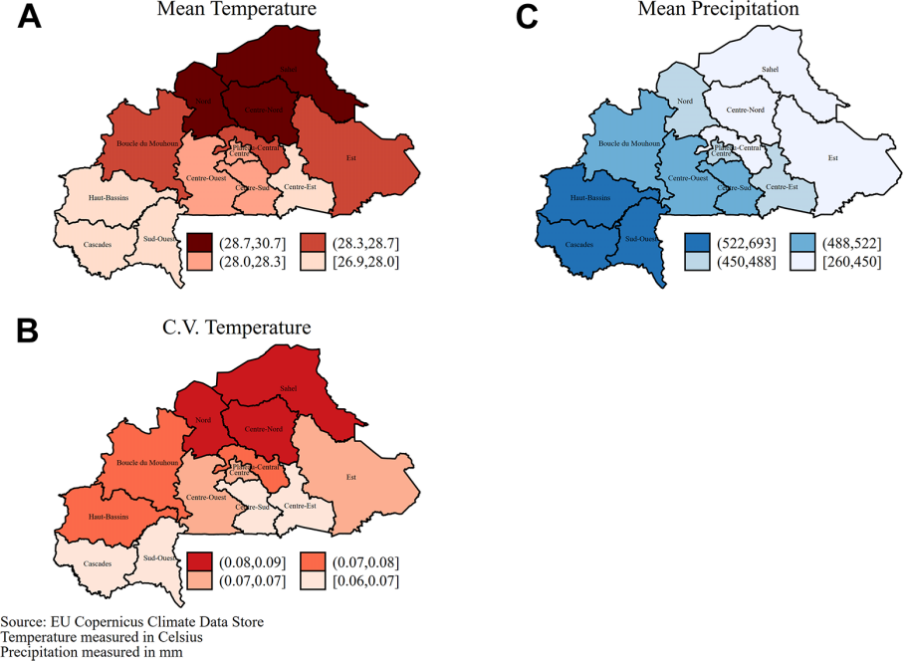
Regional variation of biophysical factors (2013 cropping season).

**Figure A2.**
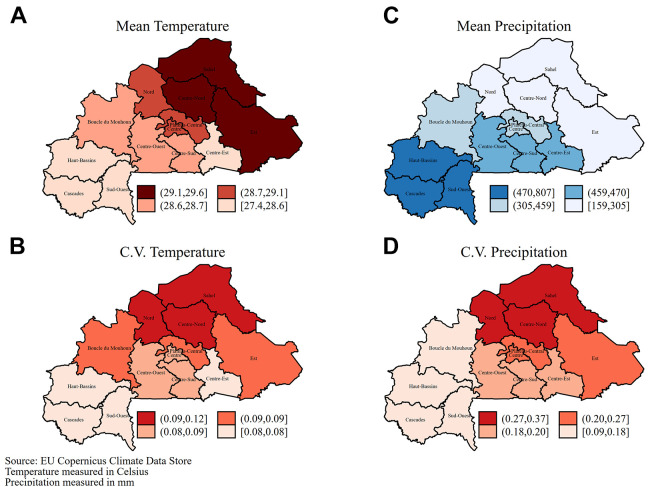
Regional variation of biophysical factors (2000-2013; monthly averages).

Figure 1 shows the spatial variations in the mean and CV of temperature (panel A and B) and precipitation (panel C and D) during the 2013 cropping season. On average, northern regions (Sahel, Centre-Nord, and Nord) had the warmest and most variable temperature while regions in the southwest (Haut-Bassins, Cascades, and Sud-Ouest) had cooler temperature and received the highest precipitation. These trends based on 2013/2014 weather data are in line with trends based on historical data on precipitation and temperature (see Figure A2). A summary of the climatic variables has been presented in Table A2.

**Figure 2.**
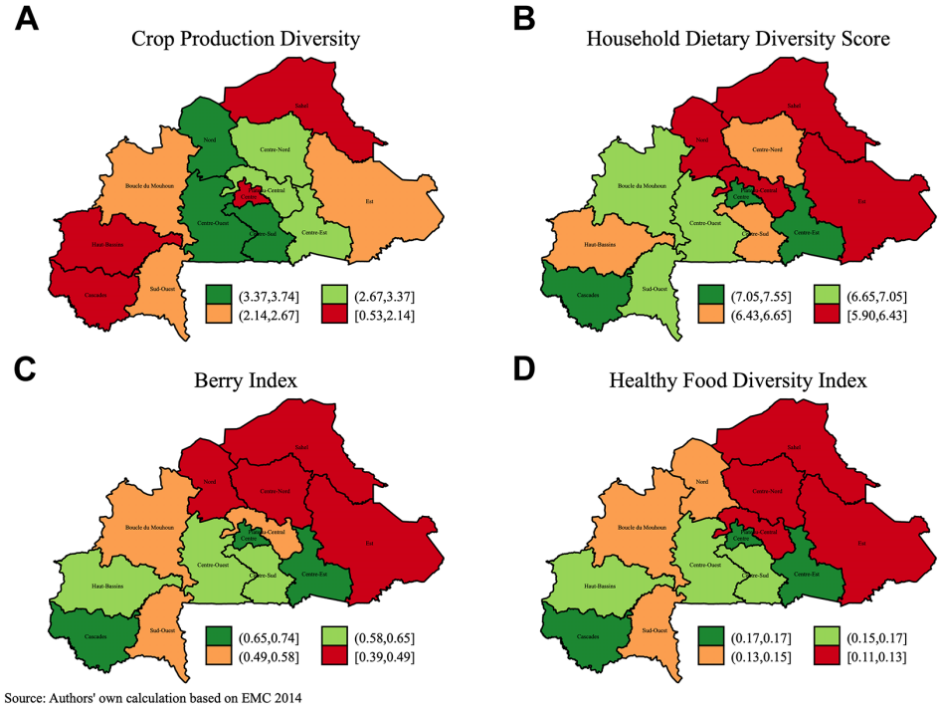
Regional variation of production and consumption.

**Figure A3.**
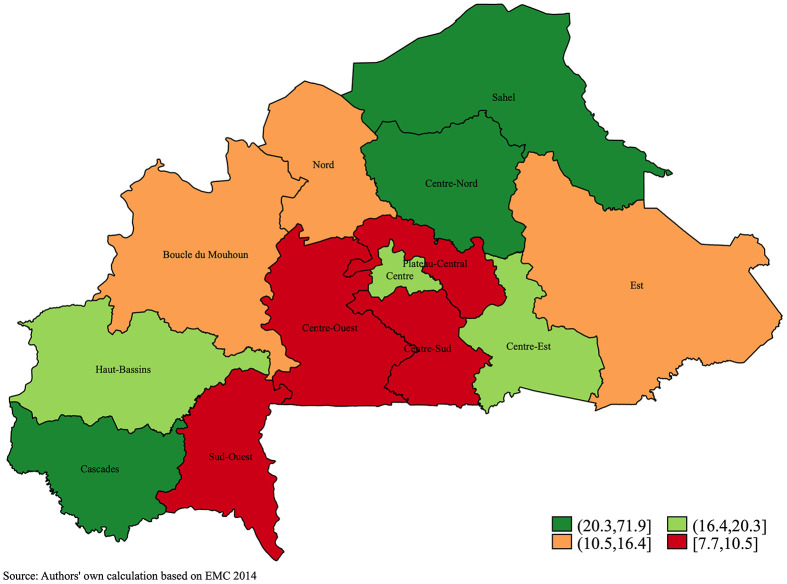
Milk consumption in the population (%), by region.

Figure 2 shows that the Nord, Center-Quest, and Center-Sud regions had the more diverse crop production (panel A), while the relatively drier Sahel region, the mostly nonagricultural Center region consisting of the capital city Ouagadougou, and the relatively wetter Haut-Bassins and Cascades regions have the least diverse crop production. The least diverse household diets are observed in Sahel and Est regions, irrespective of the diversity index used, while the ranking of Centre-Nord and Nord regions based on dietary diversity is sensitive to the diversity metric. On the other hand, Western, Center-Est, and the capital Plateau-Central regions have the most diverse diets on average. Comparing Figures 1 and 2, we note that regions with the highest average precipitation (Hauts-Bassins and Cascades) as well as that with the least precipitation (Sahel) both have the least diversified crop production. The latter is to be expected given the dominance of livestock farming in the drier Sahel region^57^ and the fact that the EMC does not have data on livestock production. High levels of land degradation^58^ has pushed households toward (agro) pastoralism, making the region the largest producer of milk in the country.^59^ The relatively high milk supply in the region may also explain why the region has the highest milk consumption (see Figure A3).

### Regression Results

Table 4 reports regression results where 2 sets of models are estimated for each dietary diversity indicator. Model (1) controls for all household-level covariates discussed in Data Sources and Variables section as well as region fixed effects. Model (2) in addition controls for climatic variables discussed in Data Sources and Variables section that may affect agricultural production. The significance and direction of parameter estimates appear to be sensitive to the choice of dietary diversity index with far fewer covariates having significant association with dietary diversity when the outcome variable is HFDI (see also the summary in Table 5). These results underscore potentially more complex interactions that determine the distribution of the quantity of food items consumed than those that determine the mere count of good groups.

**Table 4.** Regression Results.^a,b,c,d^

	HDDS		Berry Index		HFDI	
	(1)	(2)	(1)	(2)	(1)	(2)
Household size	0.0000114	0.0000846	−0.00203^e^	−0.00191^e^	0.000350^f^	0.000408^g^
	[0.02]	[0.12]	[−2.82]	[−2.67]	[1.75]	[2.07]
Urban resident	−0.00868	−0.0118	0.0727^e^	0.0706^e^	0.0111^e^	0.0108^e^
	[−1.10]	[−1.50]	[9.04]	[8.90]	[5.74]	[5.66]
Female household head	0.0373^e^	0.0352^e^	0.0158^f^	0.0134^f^	0.000652	0.000138
	[4.28]	[4.06]	[1.96]	[1.69]	[0.30]	[0.06]
Household head age	−0.0000605	−0.0000437	−0.000794^e^	−0.000770^e^	−0.0000650	−0.0000634
	[−0.32]	[−0.23]	[−4.45]	[−4.38]	[−1.38]	[−1.36]
Household head education at least primary	0.0271^e^	0.0268^e^	0.0240^e^	0.0240^e^	0.000327	0.000442
	[3.08]	[3.07]	[3.81]	[3.88]	[0.20]	[0.28]
No. of household durables owned	0.0306^e^	0.0305^e^	0.0195^e^	0.0194^e^	0.00315^e^	0.00310^e^
	[30.02]	[29.80]	[18.15]	[17.88]	[12.69]	[12.31]
No. of crop food items produced	0.0130^e^	0.0129^e^	0.00149	0.00237	0.00116^g^	0.00134^g^
	[6.09]	[6.07]	[0.71]	[1.16]	[2.14]	[2.54]
No. of unique agricultural equipment owned	0.000608	0.0000808	−0.000520	−0.00147	0.000175	−0.0000145
	[0.30]	[0.04]	[−0.25]	[−0.73]	[0.31]	[−0.03]
Travel time to market more than 15 minutes	−0.00552	−0.00657	−0.0175^e^	−0.0173^e^	−0.00230	−0.00206
	[−0.75]	[−0.90]	[−2.83]	[−2.85]	[−1.41]	[−1.27]
*2013 Cropping season*						
Total precipitation		−0.00110^e^		−0.000818^f^		−0.0000382
		[−2.86]		[−1.79]		[−0.35]
Mean monthly temperature		−0.267^e^		−0.126		0.0175
		[−2.95]		[−1.25]		[0.72]
CV monthly temperature		0.653		1.029		0.125
		[0.25]		[0.34]		[0.17]
2000-2013						
Mean total annual precipitation		0.000168		−0.0000208		−0.000131
		[0.54]		[−0.05]		[−1.34]
CV total annual precipitation		−0.688^e^		−0.523		−0.0720
		[−2.64]		[−1.60]		[−0.83]
Mean monthly temperature		0.160^f^		0.0456		−0.0382
		[1.82]		[0.46]		[−1.62]
CV monthly temperature		8.357^e^		−0.891		−2.465^e^
		[2.61]		[−0.26]		[−3.20]
Constant	1.694^e^	4.485^e^	0.556^e^	3.304^e^	0.142^e^	1.036^e^
	[105.83]	[5.15]	[39.33]	[3.60]	[39.74]	[4.06]
*R* square	-	-	0.348	0.355	0.173	0.180
Observations	10631	10631	10631	10631	10631	10631

Abbreviations: CV, coefficient of variation; HDDS, Household Dietary Diversity Score; HFDI, Healthy Food Diversity Index; OLS, ordinary least squares.

^a^ *t* statistics in brackets.

^b^ Region level fixed effects have been controlled for in all models.

^c^ Robust standard errors clustered at the enumeration zone. We run Poisson regressions for the HDDS and Food Count outcome variables; whereas for the Berry Index and HFDI, we run OLS regressions. Estimates have been weighted using the sample weight option in Stata.

^d^ Less than primary education of the head and travel time to market less than 15 minutes are the reference groups for, respectively, household head education and travel time to the nearest market variables.

^e^ *P* < .01.

^f^ *P* < .1.

^g^ *P* < .05.

**Table 5.** Summary of Regression Results by Dietary Diversity Index.^a^

Control	HDDS	Berry Index	HFDI
Household size	NS	Negative	Positive
Urban	NS	Positive	Positive
Female household headship	Positive	Positive	NS
Household head age	NS	Negative	NS
Household head education above primary	Positive	Positive	NS
Number of durables owned	Positive	Positive	Positive
Number of food items produced	Positive	NS	Positive
Number of agri. equipment owned	NS	NS	NS
Nearest market 15 or more minutes away	NS	Negative	NS
*2013 Cropping season*			
Total precipitation	Negative	Negative	NS
Mean monthly temperature	Negative	NS	NS
CV monthly temperature	NS	NS	NS
*2000-2013*			
Mean total annual precipitation	NS	NS	NS
CV total annual precipitation	Negative	NS	NS
Mean monthly temperature	Positive	NS	NS
CV monthly temperature	Positive	NS	Negative

Abbreviations: CV, coefficient of variation; HDDS, Household Dietary Diversity Score; HFDI, Healthy Food Diversity Index; NS, association not (statistically) significant.

^a^ Results are from model (2) reported in Table 4.

For example, while large household size is positively correlated with the HFDI, the association is negative when we use BI. The contrast between the BI and the HFDI may be explained by larger families choosing to concentrate their consumption on selected healthy food items. Similarly, relative to rural residents, urban residents have a more diverse diet measured by BI and HFDI, while we do not find any significant trend when considering the HDDS.

Female household headship is positively associated with dietary diversity when the HDDS and the BI are used, but not significant when diversity is measured using the HFDI. Although we are unable to draw conclusive results on the basis of the HFDI, the positive association based on the other indicators supports previous findings that highlight the importance of women’s empowerment for improved dietary and nutritional outcomes.^60,61^ Compared to households headed by individuals with less than primary education, those with household heads with at least primary education have higher dietary diversity measured by HDDS and the BI. In addition to human capital, physical capital, measured by the distinct number of durables owned, is also positively associated with dietary diversity and the result is robust to the outcome indicator used.

Production diversity expressed as the number of unique crops produced is positively correlated with the HDDS (in line with previous studies, see the studies of Jones,^34,38^ Koppmair et al,^42^ Amugsi et al^62^) and the HFDI. Interestingly, the number of agricultural equipment owned does not seem to correlate with any of our measures of dietary diversity. Households who live more than 15 minutes away from the nearest market have a less diverse diet than those who live within 14 minutes, highlighting the importance of market access as has previously been documented.^34,38,42,62^ In rural developing settings like most of Burkina Faso where food markets are often scattered, access to certain food groups (eg, fruits, vegetables, and animal-sourced foods) will likely be challenging regardless of a household’s purchasing power.

Looking at climatic variables, we observe a negative association between rainfall for 2013 and dietary diversity for all outcome variables except HFDI. Although the impact pathway is unclear, it may have been driven by the spatial variation in climatic and crop production patterns discussed in Descriptive Summary section. Areas with historically more variable rainfall also appear to have less diverse diets.

## Conclusion

This study examined the determinants of household dietary diversity in Burkina Faso using alternative indicators of dietary diversity, computed on nationally representative household survey data. We measure dietary diversity based on the oft-used HDDS and 2 less frequently used indices—the BI and the HFDI. The latter index is a considerable improvement over the HDDS since it captures both the distribution of food groups and their associated health value. Results based on the HDDS show that female household headship, household head education, asset-based household wealth, on-farm production diversity, and warmer climate all are positively associated with household dietary diversity.

Some parameter estimates are sensitive to the specific diversity index used, highlighting the need for further research to shed light on the possible sources of this difference. This limitation might potentially bias the final recommended consumption set based on food categories. Since we used food category proportions to assign health values to different food groups, it would be important to recalibrate the empirical analysis when national dietary recommendations become available. Efforts should also be made to compile health factor values used as input in the construction of the HFDI to make sure that they are relevant to the context being studied.

Localized data on food composition and health factor values are especially important in light of the nutrition transition and rapid urbanization poor countries are experiencing where diets—traditionally dominated by unprocessed staple cereals—are increasingly being replaced by animal-sourced foods and highly processed, energy-dense, and nutrient-poor plant-based foods. Given that the household survey data analyzed did not include data on livestock production, our analysis of the linkages between on-farm production and dietary diversity could be inaccurate, especially for regions with relatively high livestock wealth such as the Sahel. Given the need for integrating nutrition-sensitive strategies into agricultural development policies, consumption and expenditure surveys should always include data not only on production of crops but also of livestock and animal by-products that are essential for achieving human development potential.

Burkina Faso’s *Plan stratégique intégré de lutte contre les maladies non transmissibles* does not mention a specific strategy to promote dietary quality. In the absence of local interventions, our findings could inform policy-making by identifying possible avenues to enhance household diets. Given that urban Burkinabe households show a more diverse (and healthier) diet, concerted effort to improve availability and accessibility of food in rural Burkina Faso should be sought. The latter objective may be achieved through strategic support to production diversity in general and production of animal source foods more specifically, especially in regions with conducive climatic conditions, through supporting measures such as expansion of irrigation infrastructure.

The negative association between travel time to market and dietary diversity highlights the importance of investments on roads and transportation to create better marketing opportunities for smallholder farmers and consumers alike. More efficient and affordable marketing opportunities would increase the incentive for market-oriented farmers to produce and sell their produce, including nutrient-rich though perishable items such as vegetables and animal by-products, which in turn would increase the quantity and diversity of foods locally available.

The positive association between female headship and dietary diversity points toward the benefits of interventions that ease constraints in access to productive resources. Female Burkinabe farmers often manage plots that are relatively small and barren with limited access to inputs (eg, fertilizers, improved seeds, and draft power) and information. They also play a key role in intrahousehold decision-making on food purchases, cooking, and feeding of infants and young children, highlighting the importance of targeted behavior change communication interventions to enhance their awareness about the nutritional benefits of a diverse diet.

## Supplemental Material

Supplemental Material, sj-pdf-1-fnb-10.1177_03795721211029092 - Analyzing the Drivers of Household Dietary Diversity: Evidence from Burkina Faso Click here for additional data file. Supplemental Material, sj-pdf-1-fnb-10.1177_03795721211029092 for Analyzing the Drivers of Household Dietary Diversity: Evidence from Burkina Faso by Arkadeep Bandyopadhyay, Beliyou Haile, Carlo Azzarri and Jérôme Somé in Food and Nutrition Bulletin

## Appendix A

**Table A1.** Food Group Consumption.^a^

No.	Food group	% of households
1	Cereals Bread, corn flour, fonio, maize, maize flour, millet, millet flour, other cereal products, other cereals, pasta, rice, sorghum, sorghum flour	98
2	Roots, tubers, and plantains Cassava, potato, sweet potato, yam	16
3	Pulses, legumes, nuts, and seeds Beans, other nuts	51
4	Vegetables Greens, kapok (voaga), okra, onions, tomato paste, tomatoes	97
5	Fruits Includes pineapples, papayas, and oranges. The household was only asked about fruit consumption; therefore, we do not have data regarding the individual consumption of these items.	12
6	Meats Beef, other meats, pork, poultry, sheep/goat	45
7	Fish and seafood Dried fish, fresh fish, smoked fish	80
8	Milk and dairy products Milk, milk products	20
9	Eggs	6
10	Oils and fats Oils, other oils/greases, peanut paste, shea butter	88
11	Beverages Beer, coffee, mineral water, soft drinks, traditional beer, wine and liquors	58
12	Miscellaneous Granulated sugar, seasoning cubes, sugar cubes, sumbala seasoning, tea, kola nuts	98

^a^ Results have been weighted by survey sampling weights.

**Table A2.** Summary of Climatic Variables.^a^

Variable	Mean or %	SD	Min	Max
*Climatic variables (2013 cropping season)*				
Mean monthly temperature (°C)	28.4	0.9	26.7	32.0
CV of monthly temperature	0.1	0.0	0.1	0.1
Total precipitation (mm)	479.2	93.9	194.6	744.2
*Climatic variables (2000-2013)*				
Mean temperature (°C)	28.6	0.6	27.2	31.0
CV of temperature	0.1	0.0	0.1	0.1
Mean total annual precipitation (mm)	440.5	170.9	132.7	835.9
CV of precipitation	0.2	0.1	0.1	0.5

Abbreviations: CV, coefficient of variation; Max, maximum; Min, minimum; SD, standard deviation.

^a^ Results have been weighted by survey sampling weights.

## Note

1. The mean adequacy ratio is a member of the class of indicators used to evaluate individual intake of nutrients. This index quantifies the overall nutritional adequacy of a population based on an individual diet using the current recommended allowance for a group of nutrients of interest.

2. Estimations were ultimately run on a marginally smaller sample, since the raw data set did not have the necessary information for these omitted households.

3. The visits were made in mid-January to mid-March (postharvest season); end of April to end of June (beginning of lean season); mid-July to mid-August (end of the lean season); and finally, mid-September to mid-December (harvest season).

4. Arimond et al^54^ modeled various scenarios by constraining the maximum intake per food item at the 75th percentile of reported intake. Based on this restriction, they found that all micronutrient needs could be met with local food, although only when several nutrient dense but rarely consumed items were included in the daily diet.

5. Since the 2014 EMC did not collect data on livestock, the household production diversity score is computed based only on plant-based food groups that include the following: cereals (millets, sorghum, rice, etc); roots, tubers, and plantains (yams, taro, sweet potato, potato, etc); pulses, legumes, nuts, and seeds (peas, green beans, sesame, peanuts, etc); vegetables (amaranth, turnip, calabash, onion, eggplant, tomato, etc); fruits (watermelon, melon, etc), and miscellaneous (cloves, ginger, etc).

6. We recoded the EMC data to have 2 indicators of travel time to the nearest market: less than 15 minutes and 15 minutes or more.

7. We believe that this figure is underestimated as it only includes plant-based food and foods that may have been consumed outside the household.

8. Beverages and miscellaneous are excluded from the index calculation.
