# Supplementary material for: Analyzing the Drivers of Household Dietary Diversity: Evidence from Burkina Faso
Source: Food Nutr Bull. 2021 Sep 1;42(4):530–50. doi: 10.1177/03795721211029092 (PMC8637355; doi:10.1177/03795721211029092)
Supplement: Supplemental Material, sj-pdf-1-fnb-10.1177_03795721211029092 - Analyzing the Drivers of Household Dietary Diversity: Evidence from Burkina Faso [file sj-pdf-1-fnb-10.1177_03795721211029092.pdf]

Highlights

With large prevalence of undernutrition across most of the developing world, it is critical to analyze the factors which correlate with the consumption of a healthy and diverse diet. While there are several indicators which measure access to food and dietary diversity, often there is some dimension about diversity which is not covered. In this analysis, we draw a more complete picture of dietary diversity by using an array of dietary indicators, including the relatively sparsely used Healthy Food Diversity Index (HFDI). Although we find significant correlation among our indicators, we observe several differences in how our conditioning variables correlate with said indicators. When considering the HFDI as the outcome variable, we find that household size ($p < 0.05$), living in urban areas ($p < 0.01$), ownership of durable assets ($p < 0.01$), and crop production diversity ($p < 0.05$) all are positively correlated with the consumption of a diverse diet.
